# GenoBase: comprehensive resource database of *Escherichia coli* K-12

**DOI:** 10.1093/nar/gku1164

**Published:** 2014-11-15

**Authors:** Yuta Otsuka, Ai Muto, Rikiya Takeuchi, Chihiro Okada, Motokazu Ishikawa, Koichiro Nakamura, Natsuko Yamamoto, Hitomi Dose, Kenji Nakahigashi, Shigeki Tanishima, Sivasundaram Suharnan, Wataru Nomura, Toru Nakayashiki, Walid G. Aref, Barry R. Bochner, Tyrrell Conway, Michael Gribskov, Daisuke Kihara, Kenneth E. Rudd, Yukako Tohsato, Barry L. Wanner, Hirotada Mori

**Affiliations:** 1 Graduate School of Biological Sciences, Nara Institute of Science and Technology, Ikoma, Nara 630–0101, Japan; 2 Mitsubishi Space Software Co., LTD., 5–4–36 Tsukaguchihonnmachi, Amagasaki, Hyougo 661–0001, Japan; 3 Institute of Advanced Biosciences, Keio University, Tsuruoka 997–0017, Japan; 4 Axiohelix, Okinawa Sangyo Shien Center, 502,1831–1, Oroku, Naha-shi, Okinawa 901–0152, Japan; 5 Department of Computer Science, Purdue University, 305 N. University Street, West Lafayette, IN 47907–2107, USA; 6 Biolog, Inc., Hayward, CA 94545, USA; 7 Department of Microbiology and Plant Biology, University of Oklahoma, Norman, OK 73019–0245, USA; 8 Department of Biological Sciences, Purdue University, 915 W. State Street, West Lafayette, IN 47907–2054, USA; 9 Department Biochemistry and Molecular Biology, University of Miami, P.O. Box 016129, Miami, FL 33101–6129, USA; 10 Department of Bioinformatics, Ritsumeikan University, 1–1–1 Nojihigashi, Kusatsu, Shiga 525–8577, Japan; 11 Department of Microbiology and Immunobiology, Harvard Medical School, Boston, MA 02115, USA

## Abstract

Comprehensive experimental resources, such as ORFeome clone libraries and deletion mutant collections, are fundamental tools for elucidation of gene function. Data sets by omics analysis using these resources provide key information for functional analysis, modeling and simulation both in individual and systematic approaches. With the long-term goal of complete understanding of a cell, we have over the past decade created a variety of clone and mutant sets for functional genomics studies of *Escherichia coli* K-12. We have made these experimental resources freely available to the academic community worldwide. Accordingly, these resources have now been used in numerous investigations of a multitude of cell processes. Quality control is extremely important for evaluating results generated by these resources. Because the annotation has been changed since 2005, which we originally used for the construction, we have updated these genomic resources accordingly. Here, we describe GenoBase (http://ecoli.naist.jp/GB/), which contains key information about comprehensive experimental resources of *E. coli* K-12, their quality control and several omics data sets generated using these resources.

## INTRODUCTION

*Escherichia coli* K-12 is clearly one of the best studied organisms (1) and has had an enormous contribution on construction of concept of genes over the past half century (2). It is, however, still far from completely understood at the systems level, although complete genome structure was established and numerous functional analyses have been performed. Since the genome structure was determined, new streams of biology have come up. Omics approaches, such as cytomics (3), interactome (4), phenomics (5), proteomics (6), transcriptomics (7), etc. have been developed according to the innovation of high-throughput (HT) technologies. Simultaneously, quantitative, single cellular or single molecule analysis have emerged and become increasingly important in the field of systems biology. More and more in-depth studies of *E. coli* will no doubt continue to provide new, exciting insights into complete understanding the cell at the systems level as one of the model organisms.

To accelerate this direction, the development of biological resources for systematic studies of *E. coli* K-12, like the Keio single-gene deletion collection (8) and the ASKA ORFeome clone libraries (9), has proven especially valuable worldwide. The dramatic advent of technologies for acquisition of HT data types (e.g. the network of protein–protein interactions, transcriptional and translational regulation, genetic interaction, etc.) has created a requirement to maintain and share such resources.

The original purpose of GenoBase was to support the *E. coli* K-12 genome project launched in 1989 in Japan (10). The original data were *E. coli* K-12 sequence entries in GenBank and their mapping onto the chromosome (11) in printed format. GenoBase was designed to facilitate classification of sequenced and yet to be sequenced chromosomal regions for efficient sequencing project management for the conventional way of sequencing using Kohara-ordered phage clones (12). Following the completion of the genome project, GenoBase was enhanced to facilitate genome annotation. GenoBase originally displayed information for the W3110 strain of *E. coli* K-12 that was sequenced in the Japanese *E. coli* genome project (10,13–17), whereas the MG1655 strain, whose complete genome was first reported (18) has been more widely used. The single-gene Keio collection is derived from *E. coli* K-12 BW25113 (8) and the bar-coded deletion collection is derived from *E. coli* K-12 BW38028. Like MG1655, BW25113 and BW38028 are descendants of *E. coli* K-12 W1485. The construction of BW25113 has been reported (8).

Here, we describe key information resources available at http://ecoli.naist.jp/GB for storing, sharing and retrieving information on experimental resources of the Keio single-gene deletion collection (8), the ASKA ORFeome clone library (9) and their quality control to check duplications (19). HT screening data of protein–protein interaction (20), protein localization and phenotype analysis data using BIOLOG technology (21,22) are also stored. We also briefly summarize features of the latest version of GenoBase, which is at http://ecoli.naist.jp/GB/.

## DEVELOPMENT OF THE GenoBase SYSTEM

**Figure 1.**
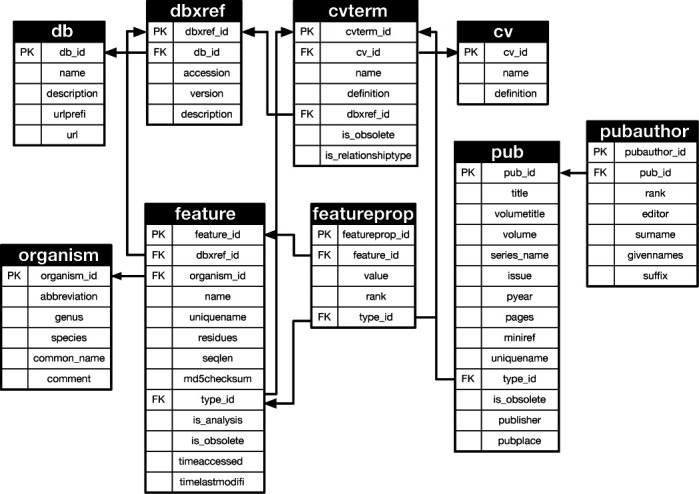
Chado schema in GenoBase. The portion of the Chado schema is shown that was used to realize the abstract GenoBase schema. Arrows show relationships between a Foreign Key (FK) and Primary Key (PK) of the same name in the database tables, except FK type_id in the feature and featureprop tables are connected to PK cvterm_id in the cvterm table.

**Figure 2.**
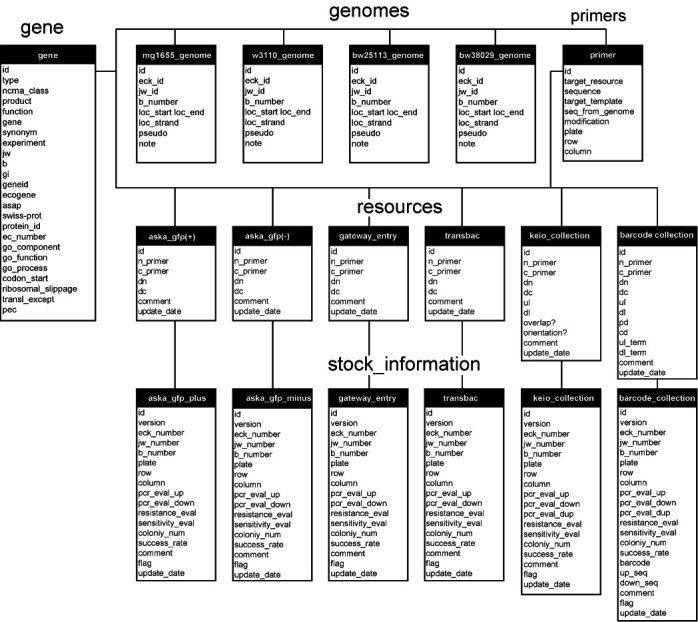
SQL Views in GenoBase. The GenoBase schema was built via SQL Views using the base relations in the Chado schema shown in Figure 1. Views and scripts are downloadable from the GenoBase description page.

Standardization is an important issue when maintaining a database for the long term. To perform this, we used the PostgreSQL database management system and the Chado schema (23,24) for numerous different types of biological data. PostgreSQL is an open source software and one of the most widely used relational database management systems. Chado is a relational database schema designed to manage biological knowledge for a wide variety of organisms. Our database was originally started as an organism-specific genome database and was previously implemented into the same relational database management system PostgreSQL with a specific schema. Here, we switched to use the Chado schema for interoperability between databases. Figure 1 shows the Chado schema used for GenoBase. The Chado schema and its associated tools were downloaded from the GMOD web site (http://www.gmod.org). Because our target organism, *E. coli* K-12, is one of the eubacteria model species, some Chado tables designed for eukaryotes were not required. Therefore, the tables in Figure 1 show a subset of those in Chado. All the data in the previous version of our database were converted and stored in the PostgreSQL database using the Chado schema and View tables (Figure 2) on the Linux operating system.

Once all the data were converted into the new database, the views of the virtual data table were defined by SQL from the Chado schema. All of the SQL scripts are downloadable from the top page links of the GenoBase website (http://ecoli.naist.jp/GB/).

## MAJOR PURPOSE OF GenoBase

**Figure 3.**
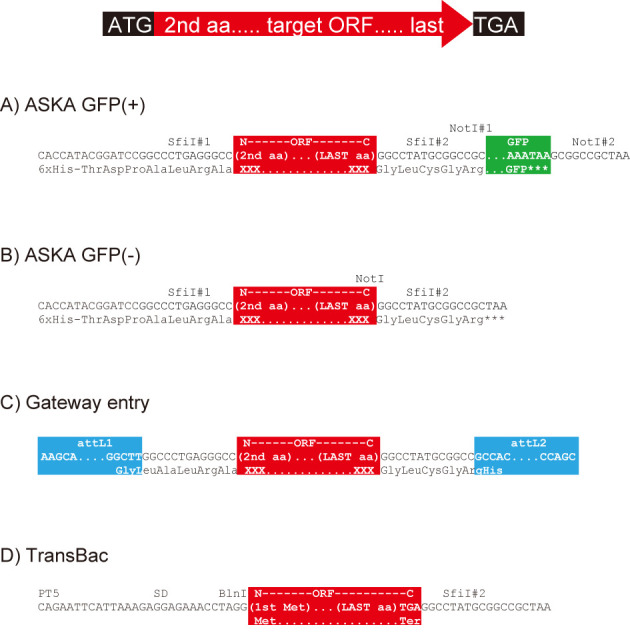
Structure of cloned region of ORF clone libraries. Colored boxes, red, green and blue boxes represent target ORF, eGFP and attL regions, respectively. All of ORF cloning target regions are designed from the second to the last amino acid coding region except the TransBac plasmid clone library.

**Figure 4.**
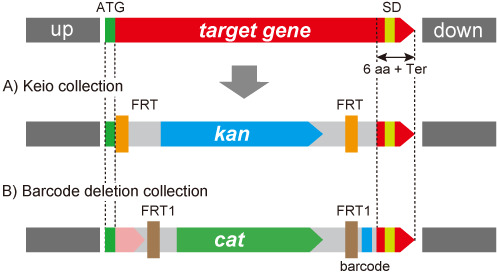
Genomic structure of target region of deletion collections. Two sets of deletion collections have been constructed using the same primer set. The deleted regions are the same between Keio and Barcode collections. FRT in the Keio and FRT1 of the Barcode collection are not site-specifically recombined by FLP recombinase.

Our database is focused on information about our comprehensively constructed experimental resources (libraries of plasmid clones, deletion mutants, etc.) and HT experimental data from a large *E. coli* functional genomics project that far exceeds all other resources combined. All the information in GenoBase is publicly available. Current resources include: four types of annotated Open Reading Frame (ORF) plasmid clone libraries and two types of deletion collections. The plasmid clone libraries include: the ASKA ORFeome libraries with (A) and without (B) a C-terminal GFP fusion (9,25), Gateway entry clone library (C; (25)) and the latest TransBac library (H. Dose, unpublished) as shown in Figure 3. The single-gene deletion collections include: the Keio collection (A; (8)) and a recently constructed barcode deletion collection (B) as shown in Figure 4. The Barcode deletion collection, whose manuscript is now in preparation, was originally constructed for the systematic analysis of synthetic lethal/sickness genetic interaction to make double genes knockout by combining single-gene deletion from the Keio and the barcode collection by conjugation (26,27, R. Takeuchi, unpublished). We added a further valuable feature, 20-nt molecular barcode, which makes population analysis and multiplex parallel screening of mixed cultures feasible (Y. Otsuka *et al.*, unpublished).

## INFORMATION ON NEW AND FUTURE RESOURCES

Information on new resources to be constructed in the future and the data from HT experiments will be disseminated, shared and publicized via GenoBase. Currently, our small RNA deletion collection with or without a barcode, chromosomal Venus-GFP fusion strains, antibodies against purified *E. coli* proteins are being prepared for dissemination.

## INFORMATION ON THE QUALITY CONTROL OF EXPERIMENTAL RESOURCES

GenoBase provides not only information about resources constructed in our lab and HT experimental data using these resources, but also information on quality control of these resources. Both the Keio and barcode single-gene deletion collections include two independent mutants of every non-essential gene. As we noted in our original report of the Keio collection (8), gene amplification during construction can result in mutants containing both the correct gene deletion and a copy of the targeted gene elsewhere. It was therefore important to validate the deletion collections not only for the presence of novel, expected junction fragments but also for absence of the targeted gene (19). By testing for absence of a genetic duplication, we verified absence of the targeted gene in at least one of the two mutants for 98.3% of the Keio collection mutants. It is notable that Giaever *et al.* (28) reported a similar percentage of yeast deletion mutants with partial duplications. Such gene amplifications have been shown to occur frequently following generalized transduction in bacteria (29–31) as well as spontaneously (32–36). Compensatory mutations can also arise during mutant construction, especially among mutants showing a deleterious effect, regardless of whether the organism is a virus, a prokaryote or eukaryote (37). To circumvent problems and misinterpretations that happen from HT screening of collections in which a small number of mutants may have duplications or compensatory mutations, we routinely examine both Keio collection mutants for a particular gene. Whenever we find ambiguous results, we construct and test additional mutants. We have found that the simplest method for construction of additional mutants is to generate polymerase chain reaction (PCR) products on the deletion mutant as template and primes flanking the deleted gene. Importantly, the introduction of PCR products for the gene deletion greater than 100 to 200 base pairs of flanking homology into a new strain harboring the Red system (38) yields huge numbers of recombinants and is far more efficient and reliable than generalized transduction (39).

Our initial resource included types of ASKA plasmid clone libraries, whose construction was based on the genome annotation of *E. coli* K-12 from 1997. Annotation policy at that time was lacking and sometimes included the wrong initiation codon. After completing the construction of the first version of the ASKA clone libraries, we participated in annotation jamborees organized by the late Monica Riley at the Marine Biological Laboratory, Woods Hole, MA, where we completed new annotations of *E. coli* K-12 MG1655 and W3110 which were based on updated and highly accurate genome sequences of *E. coli* K-12 MG1655 and W3110 (40) The resulting annotation corrections led us to re-construct ASKA clones encoding ca.1000 ORFs. Accordingly, the current ASKA plasmid clone libraries consists of more than 5000 plasmid clones.

Histories and precise information about the methods for construction of each of the resources can be downloaded from the GenoBase website (http://ecoli.naist.jp/GB/). Notably, the original sources of genomic DNAs differ for each of the resources. The ASKA plasmid clone libraries were originally constructed using the Kohara clone phages (12) as DNA sources for PCR amplification. The Gateway-fitted entry clone library was based on the ASKA plasmid clones, except for about 100 target genes, which failed to be isolated by *Sfi*I cloning from the original clones as described in detail elsewhere (25). The newly designed TransBac library follows the latest annotation of the MG1655 and *E. coli* K- BW38028 genomic DNA for PCR amplification. The Keio deletion collection mutants were using *E. coli* K-12 BW25113 as parent and the Barcode deletion collection mutants were constructed in *E. coli* K-12 BW38028. Due to use of different annotations, we computed differences between the positions of the target genes on the chromosome from the primer set and the latest annotation of GenBank. For the plasmid clone libraries, DN (Difference of N-terminus) and DC (Difference of C-terminus) were calculated as given in Figure 6 (A) and (B). For the deletion collection, in addition to DN and DC, UL (Upstream Length) and DL (Downstream Length) were determined. Also, in the case of complete overlap with the neighbor genes, PD (Partial Deletion) and CD (Complete Deletion) were evaluated as illustrated in Figure 6 (C).

**Figure 5.**
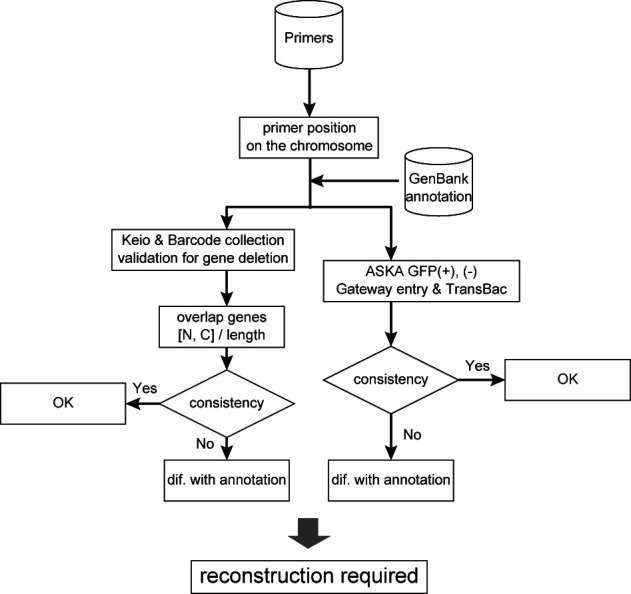
Flowchart of evaluation by the latest GenBank annotation. All of resources are generated by DNA primers, which are designed according to the latest annotation at the time of construction.

**Figure 6.**
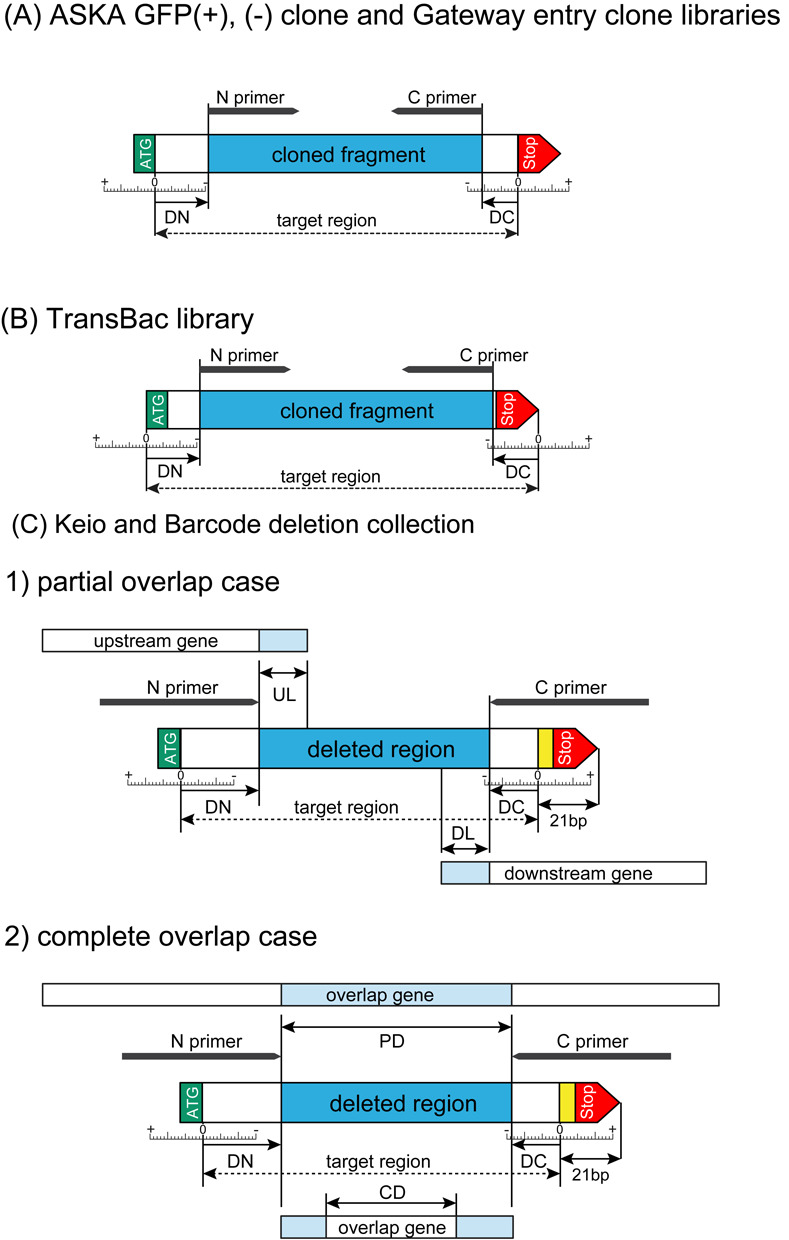
Differences with annotation. Each of resource pages for target gene shows inconsistency if it exists. (A) ASKA GFP(+), ASKA GFP(−) and Gateway entry clone validation. (B) TransBac library. (C) Keio and Barcode deletion collections.

Confirmation of the *E. coli* K-12 MG1655 and W3110 genome sequences has been previously performed (15), yet some sequencing errors may exist. Confirmation of the host strains *E. coli* K-12 BW25113 and BW38028 (BW38029 is an independent isolate) of the Keio and barcode single-deletion collections, by deep sequencing has now been completed (Y. Otsuka *et al.*, unpublished). Currently, we are completing the annotation of these strains, which will be reported elsewhere and made publicly available through GenoBase.

Changing the annotation, especially changing the gene location, is influential for making libraries. Databases, e.g. EcoGene (41), EcoCyc (42) and PEC (43) are frequently updated. We would like to keep the GenoBase experimental resources updated according to such latest information. However, in practice, it is not easy to achieve. At the least, we can calculate their inconsistency between the design and the latest annotation.

Finally, when constructing target resources, sometimes unpredicted biological events may happen, such as transposition, duplication, integration, introducing mutations, etc. Other possibilities result from the primer sequences. For the Keio collection, we performed genome confirmation by PCR amplification between antibiotic resistance fragments replaced with the target gene and upstream or downstream genes. Even though the PCR evaluation showed predicted genomic structure, we realized the existence of another wild-type copy of the target gene somewhere on the chromosome. So, we tested all of the Keio collection strains to detect such duplication, as reported elsewhere (19). In some cases, suppressor mutations may occur elsewhere on the chromosome during or after deletion or cloning. While it is possible to test such possibilities experimentally, it is technically not practical. Whether this can be achieved via community-level activities with PortEco (44) has yet to be shown. Community annotation has had success for some bacteria genera in the ASAP information resource (45,46) (http://asap.ahabs.wisc.edu/asap/home.php), but it has had limited success in the *E. coli* community.

As mentioned above, all of our resources depend on the primers designed according to the latest annotation at the time. So, we performed calculation of differences between the design and the annotation inconsistency. Evaluation scheme is shown in Figure 5.

## DATA RETRIEVAL AND CONTENTS IN GenoBase

GenoBase is a searchable web database system devoted to systems biology of *E. coli*. Querying GenoBase is done from the top page. Any word, such as an id, gene name, product name and sequence are available, and the direct searching system by SQL is also provided. Searching results are shown as a table format with links to resource pages.

**Figure 7.**
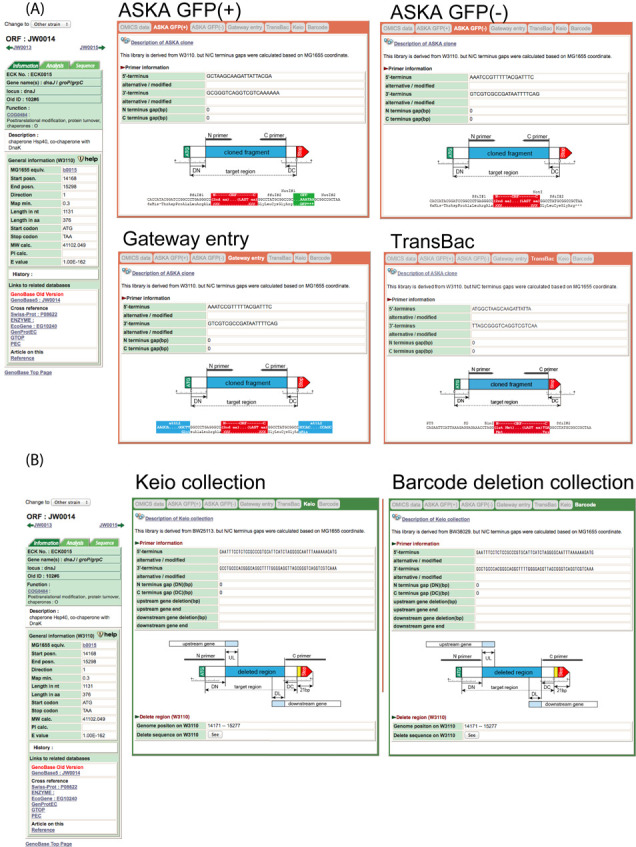

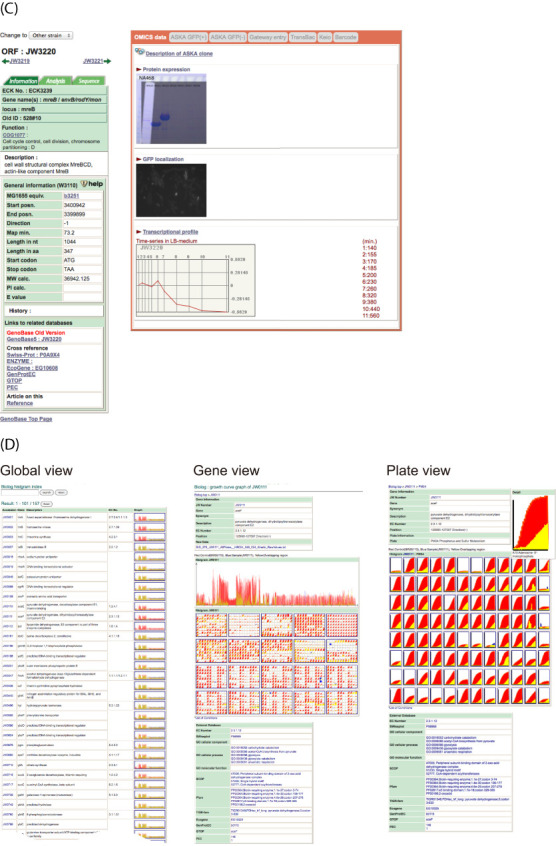
Sample screen images. (A) Sample pages of plasmid clone libraries. Schematic view of inconsistency and cloned region of target genes in nucleotide level. (B) Schematic view of deletion collection. (C) Omics results of each of the target gene. (D) BIOLOG phenotype analysis results in global, gene and plate level view.

The methods used for quality control of our resources are shown in Figure 6. On each web page, the left panel shows the information on the latest annotation basically from GenBank database entry (see Figure 7). The second and third tabs show sequences of the target genes and bioinformatics analysis of them. Currently, six tabs are available for six different resources.

**Figure 8.**
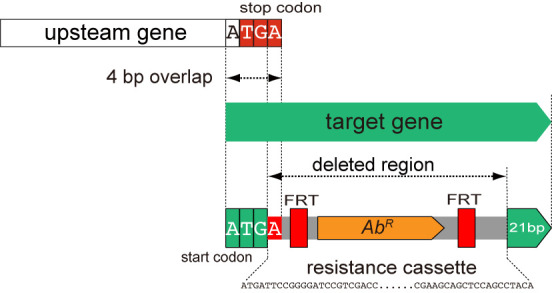
Overlap with no influence. In the case of 4 nt (ATGA) overlap between the target and the upstream genes, the termination codon of the upstream gene is re-generated in deletion strain.

For the Keio and the Barcode deletion collection, in the case of four base overlap with the upstream gene, special case has no influence for the correct termination of the upstream gene shown in Figure 8.

Another tab shows omics output using our resources. Systematic experimental data currently includes three data sets. Protein–protein interaction data are based on using His-tagged ASKA ORF clone library without GFP (20). All of the interaction data including data produced by Time of Flight Mass Spectrometry (TOF-MASS) analysis are stored and specific partner candidates as prey proteins are available from each target protein as bait.

DNA microarray analysis of about 150 single-gene deletion mutants, mostly for ones lacking transcription factors, quantified by ImaGene for deletion mutants are also stored. These data are downloadable from the link as tab-limited text format files.

Images of protein localization analyzed by the GFP-tagged ASKA ORFeome clones are available. *E. coli* cells, without isopropyl-beta-D-thiogalactopyranoside (IPTG) in Luria-Bertani broth (LB) to avoid misfolding, were analyzed by fluorescent microscopy. Images captured with a charge-coupled device (CCD) camera are also available.

Phenotype microarray analysis by BIOLOG plates (47,48) are shown graphically. Currently, about 300 data sets using single-gene knockout of the Keio collection are available (21,22). Our BIOLOG phenotype screening data have been produced by 10 times wild-type strain tests as a control and duplicate tests for each of target gene deletion strains. The screenings were performed using PM1 to PM20 both for metabolic and chemical sensitivity tests (http://www.biolog.com/pmMicrobialCells.shtml).

Other omics type results will be added and made downloadable from the GenoBase system when we obtain them. Currently, comprehensive genetic interaction data, population dynamics using the Barcode deletion collection, in addition to the simple growth condition measured by our latest colony quantification system (49) are scheduled to be opened.

## RESOURCE DISTRIBUTION

Current official distribution site is NIG (the National Institute of Genetics, Mishima http://www.shigen.nig.ac.jp/ecoli/strain/), supported by the National Bioresource Project (40).

Currently, we have four types of predicted ORF plasmid clone libraries and two of them have been distributed from NIG. The Gateway entry clone library will start soon to distribute to the academy side. We also have efforts to open our new resources timely as much as possible.

For deletion construct, only Keio collection (8) is now available from NIG and we would like to make the Barcode deletion collection publicly available as soon as possible.

## POLICY OF THE MANAGEMENT OF GenoBase

Assignment of annotation onto the genome is not our main task. The annotation information on genes depends on GenBank database entry. The major purpose of GenoBase is to provide information related to resources constructed by our group for the community to investigate *E. coli* K-12 as a model cell system. We are also producing omics data to understand what a cell system is. We hope experimental resources, their information and omics results from these resources may contribute in the community using *E. coli* as a model system.

## References

[1] Thomason M.K., Bischler T., Eisenbart S.K., Forstner K.U., Zhang A., Herbig A., Nieselt K., Sharma C.M., Storz G. (2014). Global transcriptional start site mapping using dRNA-seq reveals novel antisense RNAs in *Escherichia coli*. J. Bacteriol.

[2] Brenner S., Jacob F., Meselson M. (1961). An unstable intermediate carrying information from genes to ribosomes for protein synthesis. Nature.

[3] Jehmlich N., Hubschmann T., Gesell Salazar M., Volker U., Benndorf D., Muller S., von Bergen M., Schmidt F. (2010). Advanced tool for characterization of microbial cultures by combining cytomics and proteomics. Appl. Microbiol. Biotechnol..

[4] Rajagopala S.V., Sikorski P., Kumar A., Mosca R., Vlasblom J., Arnold R., Franca-Koh J., Pakala S.B., Phanse S., Ceol A. (2014). The binary protein-protein interaction landscape of *Escherichia coli*. Nat. Biotechnol..

[5] Nakahigashi K., Toya Y., Ishii N., Soga T., Hasegawa M., Watanabe H., Takai Y., Honma M., Mori H., Tomita M. (2009). Systematic phenome analysis of *Escherichia coli* multiple-knockout mutants reveals hidden reactions in central carbon metabolism. Mol. Syst. Biol..

[6] Martorana A.M., Motta S., Di Silvestre D., Falchi F., Deho G., Mauri P., Sperandeo P., Polissi A. (2014). Dissecting *Escherichia coli* outer membrane biogenesis using differential proteomics. PloS One.

[7] Conway T., Creecy J.P., Maddox S.M., Grissom J.E., Conkle T.L., Shadid T.M., Teramoto J., San Miguel P., Shimada T., Ishihama A. (2014). Unprecedented high-resolution view of bacterial operon architecture revealed by RNA sequencing. mBio.

[8] Baba T., Ara T., Hasegawa M., Takai Y., Okumura Y., Baba M., Datsenko K.A., Tomita M., Wanner B.L., Mori H. (2006). Construction of *Escherichia coli* K-12 in-frame, single-gene knockout mutants: the Keio collection. Mol. Syst. Biol..

[9] Kitagawa M., Ara T., Arifuzzaman M., Ioka-Nakamichi T., Inamoto E., Toyonaga H., Mori H. (2005). Complete set of ORF clones of *Escherichia coli* ASKA library (A Complete Set of E. coli K-12 ORF Archive): Unique Resources for Biological Research. DNA Res..

[10] Yura T., Mori H., Nagai H., Nagata T., Ishihama A., Fujita N., Isono K., Mizobuchi K., Nakata A. (1992). Systematic sequencing of the *Escherichia coli* genome: analysis of the 0–2.4 min region. Nucleic Acids Res..

[11] Rudd K.E. (1998). Linkage map of *Escherichia coli* K-12, edition 10: the physical map. Microbiol. Mol. Biol. Rev..

[12] Kohara Y., Akiyama K., Isono K. (1987). The physical map of the whole *E. coli* chromosome: application of a new strategy for rapid analysis and sorting of a large genomic library. Cell.

[13] Aiba H., Baba T., Hayashi K., Inada T., Isono K., Itoh T., Kasai H., Kashimoto K., Kimura S., Kitakawa M. (1996). A 570-kb DNA sequence of the *Escherichia coli* K-12 genome corresponding to the 28.0–40.1 min region on the linkage map. DNA Res..

[14] Fujita N., Mori H., Yura T., Ishihama A. (1994). Systematic sequencing of the *Escherichia coli* genome: analysis of the 2.4–4.1 min (110,917–193,643 bp) region. Nucleic Acids Res..

[15] Hayashi K., Morooka N., Yamamoto Y., Fujita K., Isono K., Choi S., Ohtsubo E., Baba T., Wanner B.L., Mori H. (2006). Highly accurate genome sequences of *Escherichia coli* K-12 strains MG1655 and W3110. Mol. Syst. Biol..

[16] Itoh T., Aiba H., Baba T., Hayashi K., Inada T., Isono K., Kasai H., Kimura S., Kitakawa M., Kitagawa M. (1996). A 460-kb DNA sequence of the *Escherichia coli* K-12 genome corresponding to the 40.1–50.0 min region on the linkage map. DNA Res..

[17] Yamamoto Y., Aiba H., Baba T., Hayashi K., Inada T., Isono K., Itoh T., Kimura S., Kitagawa M., Makino K. (1997). Construction of a contiguous 874-kb sequence of the *Escherichia coli* -K12 genome corresponding to 50.0–68.8 min on the linkage map and analysis of its sequence features. DNA Res..

[18] Blattner F.R., Plunkett G., Bloch C.A., Perna N.T., Burland V., Riley M., Collado-Vides J., Glasner J.D., Rode C.K., Mayhew G.F. (1997). The complete genome sequence of *Escherichia coli* K-12. Science.

[19] Yamamoto N., Nakahigashi K., Nakamichi T., Yoshino M., Takai Y., Touda Y., Furubayashi A., Kinjyo S., Dose H., Hasegawa M. (2009). Update on the Keio collection of *Escherichia coli* single-gene deletion mutants. Mol. Syst. Biol..

[20] Arifuzzaman M., Maeda M., Itoh A., Nishikata K., Takita C., Saito R., Ara T., Nakahigashi K., Huang H.C., Hirai A. (2006). Large-scale identification of protein-protein interaction of *Escherichia coli* K-12. Genome Res..

[21] Tohsato Y., Baba T., Mazaki Y., Ito M., Wanner B.L., Mori H. (2010). Environmental dependency of gene knockouts on phenotype microarray analysis in *Escherichia coli*. J. Bioinform. Comput. Biol..

[22] Tohsato Y., Mori H. (2008). Phenotype profiling of single gene deletion mutants of *E. coli* using Biolog technology. Genome Inform..

[23] Zhou P., Emmert D., Zhang P. (2006). Using Chado to store genome annotation data. Curr. Protoc. Bioinformat..

[24] Mungall C.J., Emmert D.B. (2007). A Chado case study: an ontology-based modular schema for representing genome-associated biological information. Bioinformatics.

[25] Rajagopala S.V., Yamamoto N., Zweifel A.E., Nakamichi T., Huang H.K., Mendez-Rios J.D., Franca-Koh J., Boorgula M.P., Fujita K., Suzuki K. (2010). The *Escherichia coli* K-12 ORFeome: a resource for comparative molecular microbiology. BMC Genom..

[26] Butland G., Babu M., Diaz-Mejia J.J., Bohdana F., Phanse S., Gold B., Yang W., Li J., Gagarinova A.G., Pogoutse O. (2008). eSGA: *E. coli* synthetic genetic array analysis. Nat. Methods.

[27] Typas A., Nichols R.J., Siegele D.A., Shales M., Collins S.R., Lim B., Braberg H., Yamamoto N., Takeuchi R., Wanner B.L. (2008). High-throughput, quantitative analyses of genetic interactions in *E. coli*. Nat. Methods.

[28] Giaever G., Chu A.M., Ni L., Connelly C., Riles L., Veronneau S., Dow S., Lucau-Danila A., Anderson K., Andre B. (2002). Functional profiling of the *Saccharomyces cerevisiae* genome. Nature.

[29] Hill C.W., Schiffer D., Berg P. (1969). Transduction of merodiploidy: induced duplication of recipient genes. J. Bacteriol..

[30] Hill C.W., Foulds J., Soll L., Berg P. (1969). Instability of a missense suppressor resulting from a duplication of genetic material. J. Mol. Biol..

[31] Anderson R.P., Miller C.G., Roth J.R. (1976). Tandem duplications of the histidine operon observed following generalized transduction in *Salmonella typhimurium*. J. Mol. Biol..

[32] Anderson R.P., Roth J.R. (1977). Tandem genetic duplications in phage and bacteria. Ann. Rev. Microbiol..

[33] Anderson R.P., Roth J.R. (1978). Tandem chromosomal duplications in *Salmonella typhimurium*: fusion of histidine genes to novel promoters. J. Mol. Biol..

[34] Anderson R.P., Roth J.R. (1978). Tandem genetic duplications in *Salmonella typhimurium*: amplification of the histidine operon. J. Mol. Biol..

[35] Anderson R.P., Roth J.R. (1979). Gene duplication in bacteria: alteration of gene dosage by sister-chromosome exchanges. Cold Spring Harb. Symp. Quant. Biol..

[36] Anderson P., Roth J. (1981). Spontaneous tandem genetic duplications in *Salmonella typhimurium* arise by unequal recombination between rRNA (rrn) cistrons. Proc. Natl. Acad. Sci. U.S.A..

[37] Poon A., Davis B.H., Chao L. (2005). The coupon collector and the suppressor mutation: estimating the number of compensatory mutations by maximum likelihood. Genetics.

[38] Datsenko K.A., Wanner B.L. (2000). One-step inactivation of chromosomal genes in *Escherichia coli* K-12 using PCR products. Proc. Natl. Acad. Sci. U.S.A..

[39] Zhou L., Lei X.H., Bochner B.R., Wanner B.L. (2003). Phenotype microarray analysis of *Escherichia coli* K-12 mutants with deletions of all two-component systems. J. Bacteriol..

[40] Riley M., Abe T., Arnaud M.B., Berlyn M.K., Blattner F.R., Chaudhuri R.R., Glasner J.D., Horiuchi T., Keseler I.M., Kosuge T. (2006). *Escherichia coli* K-12: a cooperatively developed annotation snapshot–2005. Nucleic Acids Res..

[41] Zhou J., Rudd K.E. (2013). EcoGene 3.0. Nucleic Acids Res..

[42] Keseler I.M., Mackie A., Peralta-Gil M., Santos-Zavaleta A., Gama-Castro S., Bonavides-Martinez C., Fulcher C., Huerta A.M., Kothari A., Krummenacker M. (2013). EcoCyc: fusing model organism databases with systems biology. Nucleic Acids Res..

[43] Yamazaki Y., Niki H., Kato J. (2008). Profiling of *Escherichia coli* chromosome database. Methods Mol. Biol..

[44] Hu J.C., Sherlock G., Siegele D.A., Aleksander S.A., Ball C.A., Demeter J., Gouni S., Holland T.A., Karp P.D., Lewis J.E. (2014). PortEco: a resource for exploring bacterial biology through high-throughput data and analysis tools. Nucleic Acids Res..

[45] Glasner J.D., Rusch M., Liss P., Plunkett G., Cabot E.L., Darling A., Anderson B.D., Infield-Harm P., Gilson M.C., Perna N.T. (2006). ASAP: a resource for annotating, curating, comparing, and disseminating genomic data. Nucleic Acids Res..

[46] Glasner J.D., Liss P., Plunkett G., Darling A., Prasad T., Rusch M., Byrnes A., Gilson M., Biehl B., Blattner F.R. (2003). ASAP, a systematic annotation package for community analysis of genomes. Nucleic Acids Res..

[47] Bochner B.R. (2003). New technologies to assess genotype-phenotype relationships. Nat. Rev. Genet..

[48] Bochner B.R., Gadzinski P., Panomitros E. (2001). Phenotype microarrays for high-throughput phenotypic testing and assay of gene function. Genome Res..

[49] Takeuchi R., Tamura T., Nakayashiki T., Tanaka Y., Muto A., Wanner B.L., Mori H. (2014). Colony-live -a high-throughput method for measuring microbial colony growth kinetics- reveals diverse growth effects of gene knockouts in *Escherichia coli*. BMC Microbiol..

